# Tracking EBV-encoded RNAs (EBERs) from the nucleus to the excreted exosomes of B-lymphocytes

**DOI:** 10.1038/s41598-018-33758-4

**Published:** 2018-10-18

**Authors:** Waqar Ahmed, Saeed Tariq, Gulfaraz Khan

**Affiliations:** 1 Department of Microbiology and Immunology, College of Medicine and Health Sciences, United Arab Emirates University, Al Ain, United Arab Emirates; 2 Department of Anatomy, College of Medicine and Health Sciences, United Arab Emirates University, Al Ain, United Arab Emirates

## Abstract

Epstein-Barr virus-encoded RNAs (EBER1 and EBER2) are two highly abundant, non-protein coding RNAs consistently expressed in all EBV infected cells, but their function remains poorly understood. Conventional *in situ* hybridization studies have indicated that these RNAs are present exclusively in the nucleus. We have recently demonstrated that EBERs can be excreted from infected cells via exosomes. However, the details of the steps involved in their excretion remain unknown. In this study, we aimed to directly track the journey of EBERs from the nucleus to the excretory exosomes of EBV immortalized B-lymphocytes. Using a combination of molecular and novel immuno-gold labelled electron microscopy (EM) based techniques, we demonstrate the presence of EBERs, not only in the nucleus, but also in the cytoplasm of EBV infected B cell lines. EBERs were also seen in exosomes shed from infected cells along with the EBER binding protein La. Our results show, for the first time, that at least a proportion of EBERs are transported from the nucleus to the cytoplasm where they appear to be loaded into multi-vesicular bodies for eventual excretion via exosomes.

## Introduction

Epstein-Barr virus (EBV) is an oncogenic herpesvirus aetiologically linked to several human malignancies, including Burkitt’s lymphoma, Hodgkin’s lymphoma, nasopharyngeal carcinoma and gastric carcinoma^1^. The virus is known to infect and establish life-long latency in memory B cells^2,3^. During the viral life cycle it undergoes four successive latency programs (III-II-I-0) in which different sets of viral genes are expressed. One of the transcripts that is expressed in all forms of latency are the Epstein-Barr virus encoded RNAs, EBER1 and EBER2^4^. These abundantly expressed, non-polyadenylated and non-protein coding small RNA molecules (~170 nt in length) are transcribed by RNA polymerase III. Due to their high level of expression (~10^6^ copies/cell), they have been used as targets for the detection of EBV in histological samples using *in situ* hybridization^5,6^. At the sequence level, EBERs share only 54% homology, but are highly conserved structurally and they have a well-defined secondary structure comprising of intermolecular base-pairing and several stem-loops^7,8^. EBERs have also been shown to be associated with a number of cellular proteins including the lupus antigen La^9,10^.

EBERs are typically found in the nucleus of EBV infected cells^11^. However, using highly sensitive techniques such as laser confocal microscopy, EBERs were shown to be present in the cytoplasm^12^. In fact, EBERs were initially discovered as a ribonucleoprotein complexes using anti-La antibody from the serum of systemic lupus erythematosus (SLE) patients^9^. La is an abundant cellular protein that binds to virtually all precursor RNA polymerase III transcripts^13^. This protein is mainly localized in the nucleus, but under certain conditions, a fraction of the protein can also be found in the cytoplasm^11,14^. La protein has also been shown to be secreted outside the cell via nanovesicles called exosomes^15^. Exosomes are extracellular nanovesicles (30–150 nm in size) that are generated through the membrane invaginations of multivesicular bodies (MVB)^16^. These MVBs finally result in the formation of the intraluminal vesicles (ILVs) that on release are referred to as exosomes. Exosomes have been shown to contain diverse bioactive cellular cargo, including microRNA (miRNA), DNA, proteins and even virus particles^17,18^. Moreover, the EBER-binding cellular protein La, has also been shown to be present in exosomes^15^, suggesting that EBERs could ‘piggyback’ onto La and be excreted out of the infected cells by this mechanism^10^. Exosomes carrying EBERs could be taken up by surrounding cells and influence the local microenvironment in favor of the virus^19^. Indeed, a number of studies have reported that viral infected cells, including EBV infection, can manipulate the local microenvironment by releasing exosomes containing viral and cellular components^20–22^.

We have recently demonstrated that EBER1 and EBER2 are consistently found in exosomal fractions of EBV immortalized B-cell lines and EBER transfected epithelial cells, together with the cellular protein La^23^. However, the details of the steps involved in their transport from the nucleus to the cytoplasm and finally out of the cell via exosomes remain unknown. In this study, we aimed to directly track the journey of EBERs using electron microscopy (EM) based methods. We established EM EBER *in situ* hybridization and immuno-EM for La, and applied these techniques to track EBERs at the cellular and exosomal levels. Additionally, we used qPCR to determine the relative quantities of EBER1 and EBER2 in infected cells and in their exosomal fractions.

## Results

### Localization of EBERs and La in the nucleus and cytoplasm of EBV infected B-cells

**Figure 1:**
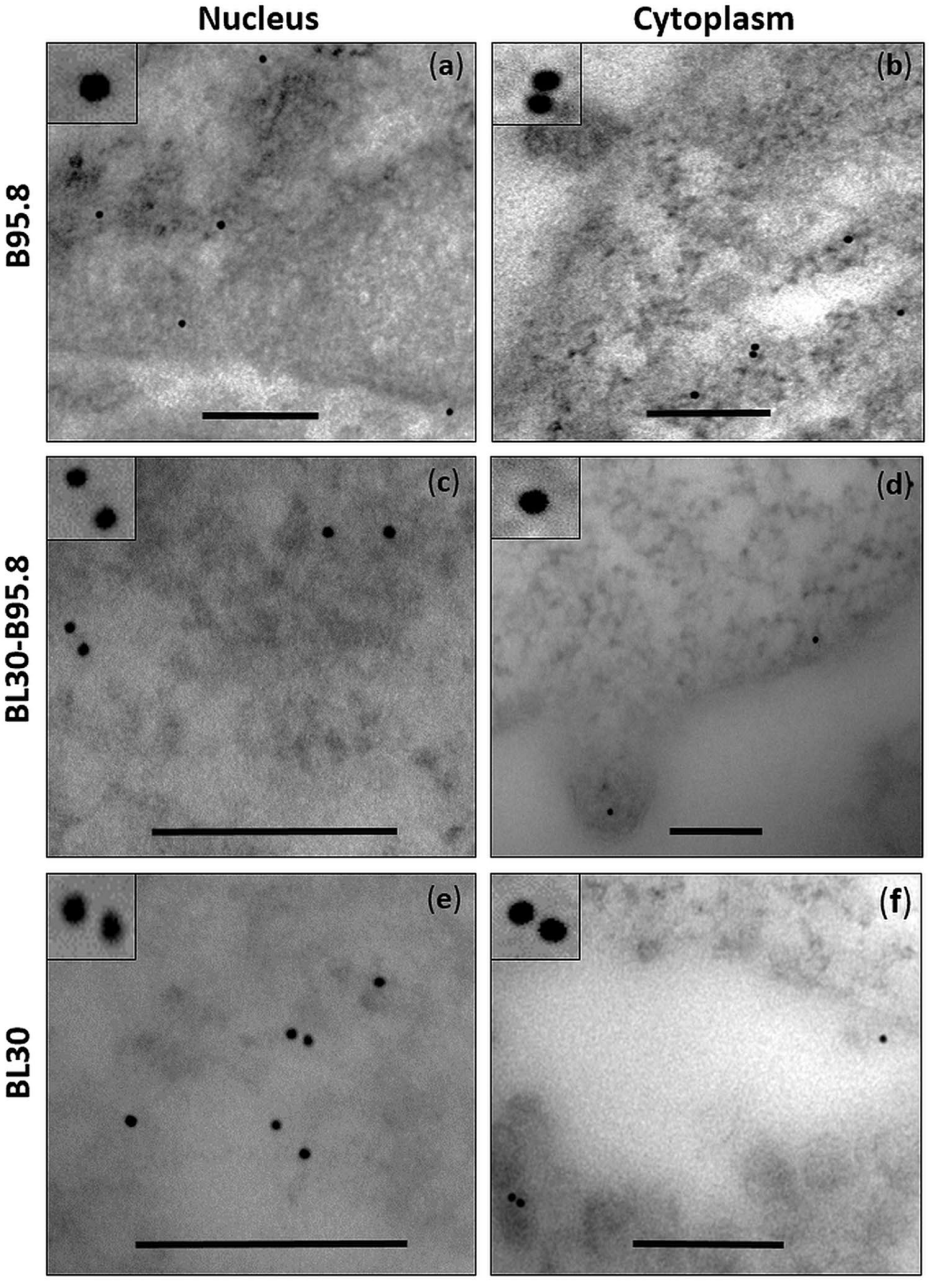
Immuno-electron microscopy staining for La protein in cells. Ultra-thin section of EBV infected (B95.8 and BL30-B95.8) and non-infected (BL30) cells were generated and stained for La using La-specific primary monoclonal antibody and 10 nm gold labelled secondary antibody. La staining was observed in the nucleus and cytoplasm of all the cells (**a**–**f**). Scale bar = 200 nm.

**Figure 2:**
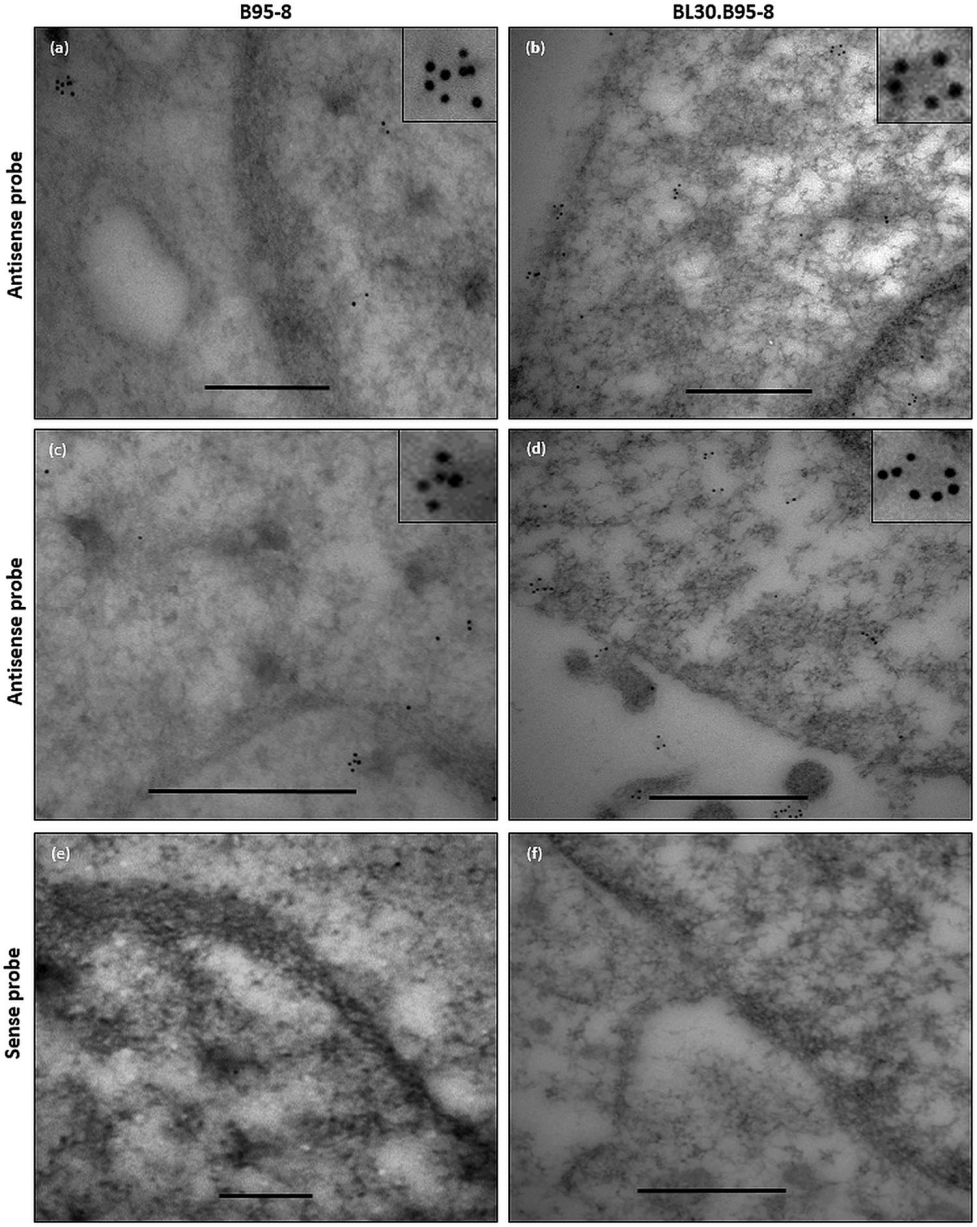
Electron microscopy *in situ* hybridization for EBERs in EBV infected cells. EM EBER *in situ* hybridization was carried out on the ultra-thin sections of B95.8 and BL30-B95.8 cells. Specific EBER signals were detected using EBER anti-sense probes and 10 nm gold labelled secondary antibody (**a**–**d**). No signals were observed using EBER sense probe (**e**,**f**). EBER signals were observed in both the nucleus and cytoplasm of the infected cells. Scale bar = 500 nm.

Indirect molecular studies have indicated that EBERs and the EBER-binding protein La are present in exosomal fractions of EBV infected cells^23–25^. To become part of the exosomal cargo, we hypothesized that a fraction of the EBERs and La must be present in the cytoplasm of the EBV infected cells. To verify this, we performed immuno-electron microscopy for La (immuno-EM La) and EM *in situ* hybridization for EBERs (EM EBER-ISH) on ultra-thin sections of EBV infected (B95.8 and BL30-B95.8) and non-infected (BL30) B-cell lines. The results indicated that La was mainly present in the nucleus but a fraction of the protein was also seen in the cytoplasm of the cells (Fig. 1). As La is an essential cellular protein, it was detected in both, EBV infected and non-infected cells. Interestingly we also observed the presence of La in vesicular bodies budding from the plasma membrane (Fig. 1d). Similarly, using EM EBER-ISH, EBERs were also seen in both, the nucleus and cytoplasm of EBV infected cells (Figs 2 and S1). By contrast, using EBER sense probes, no signals were seen in either the nucleus or cytoplasm (Figs 2 and S1).

### Purification of exosomes from EBV infected and non-infected B-cells

**Figure 3:**
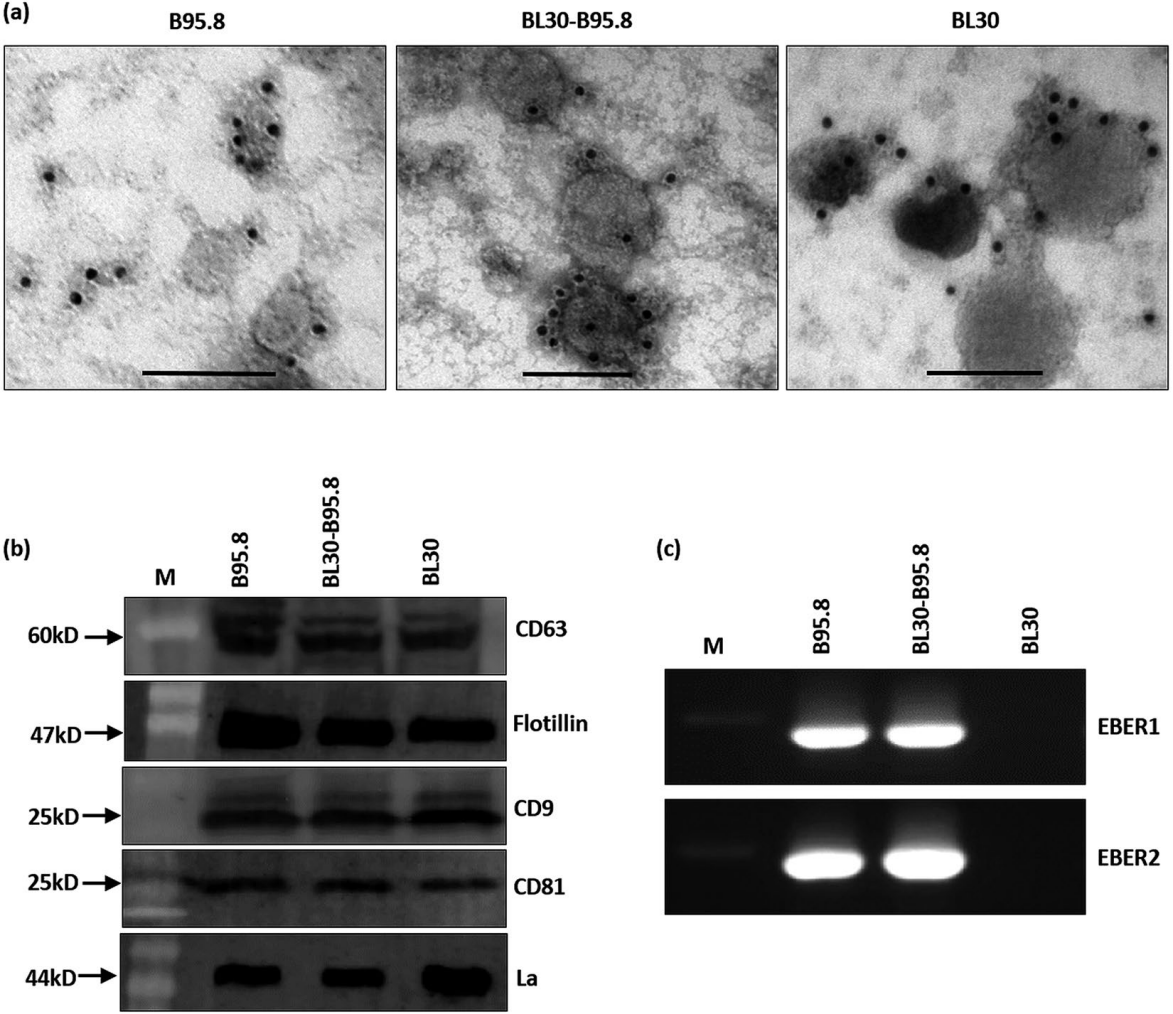
Isolation and characterization of exosomes released by EBV infected and non-infected cells. (**a**) Immuno-electron microscopy staining for exosomal marker CD63 using 10 nm gold labelled secondary antibody. Staining pattern indicated membrane association of CD63 on isolated exosomes in all cell lines (B95.8, BL30-B95.8, BL30). Scale bar = 100 nm. (**b**) Western blot for different exosomal markers and EBER-binding protein La in isolated exosomes. (**c**) RT-PCR showing the presence of EBERs in exosomes isolated from EBV infected cells (B95.8 and BL30-B95.8).

Exosomes from EBV infected (B95.8, BL30-B95.8) and non-infected (BL30) B-cell lines were isolated using differential ultracentrifugation. The identity of the isolated exosomes was first confirmed by transmission electron microscopy (TEM) and then by immuno-EM for CD63. The isolated exosomes from all 3 cell lines showed a morphology typical of exosomes (cup or disk shape) and ranged from 30–150 nm in size (Fig. S2). Immuno-EM for CD63 showed an accumulation of gold nano particles primarily on the membranes of isolated exosomes (Fig. 3a). To further confirm the identity of the isolated exosomes, western blot was performed for the exosomal markers, flotillin, CD9, CD81 and CD63. The blots clearly showed the presence of these markers in isolated exosomes (Fig. 3b). Furthermore, we also observed specific bands for La protein in the immunoblots of isolated exosomal fractions as well as the presence of both EBERs using RT-PCR (Fig. 3b,c).

### EBERs are present in exosomes of EBV infected B-cells

**Figure 4:**
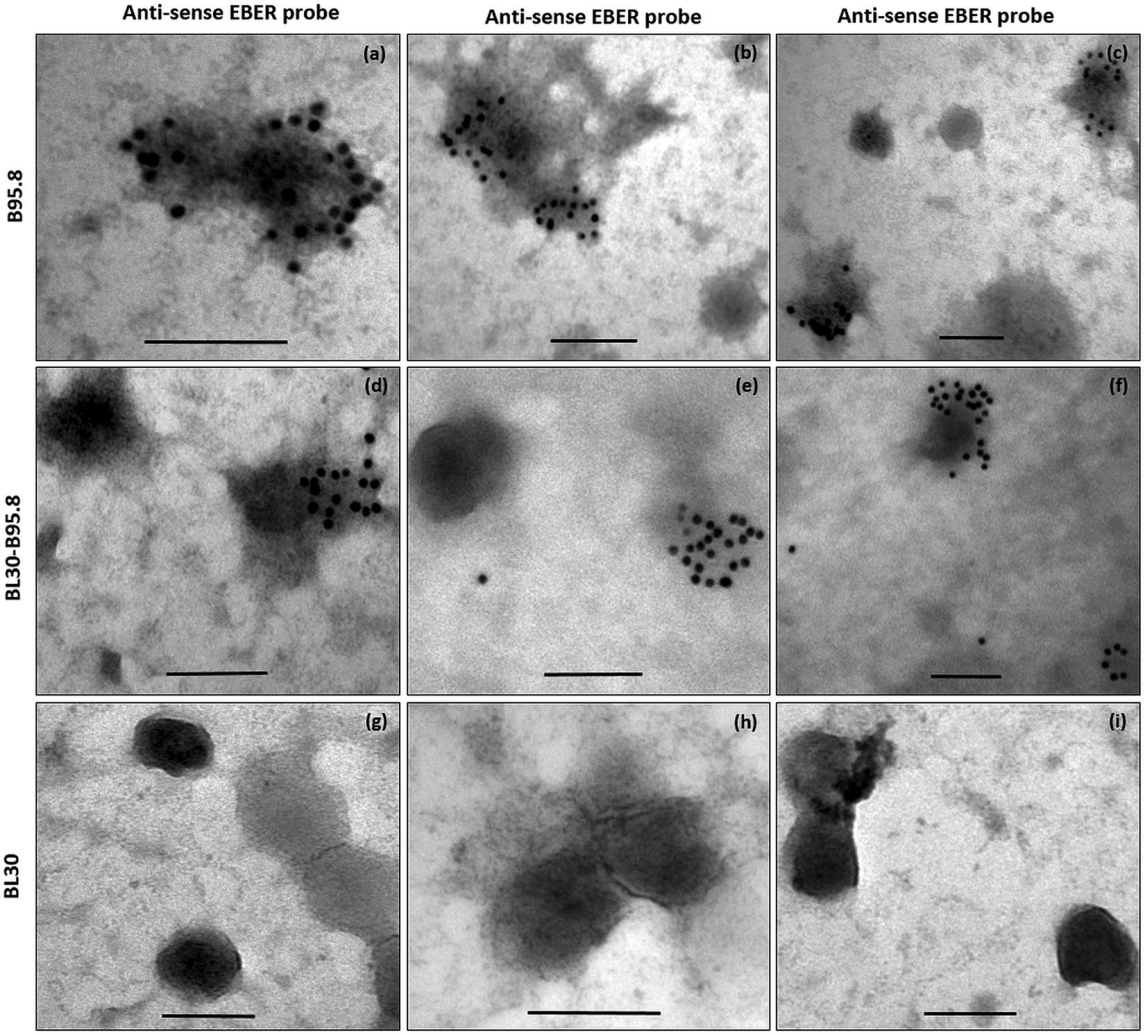
Electron microscopy *in situ* hybridization for EBERs in exosomes. Exosomes were fixed overnight in 4% paraformaldehyde and electron microscopy *in situ* hybridization was performed using 10 nm gold labelled secondary antibody. EBER staining was observed in EBV infected cells (B95.8 and BL30-B95.8) (**a**–**f**), but not in non-infected cells (BL30) (**g**–**i**). EBER staining was clearly localized to the exosomes. Scale bar = 100 nm.

A previous study reported that, both EBERs and La are present in exosomal fractions^23^. We confirmed these findings by demonstrating the presence of La protein and EBERs using western blot and RT-PCR, respectively (Fig. 3b/c). To assess whether EBERs are present inside the exosomes or in extra exosomal fractions, we performed EM EBER-ISH on isolated exosomes. Our results showed the presence of EBER signals within the exosomes of both EBV infected cells lines (B95.8 and BL30-B95.8) rather than in the extra-exosomal fractions (Fig. 4a–f). Due to the harsh conditions of the protocol, the overall morphology of the exosomes was not always well maintained and some signals associated with disrupted membranes were also observed. No EBER staining was however observed in the exosomes isolated from EBV non-infected cells (BL30 cells) (Fig. 4g–i). Moreover, the staining pattern indicated that significant amount of EBERs were excreted within each exosome.

### EBER1 and EBER2 are both released in exosomes from EBV infected B-cells

**Figure 5:**
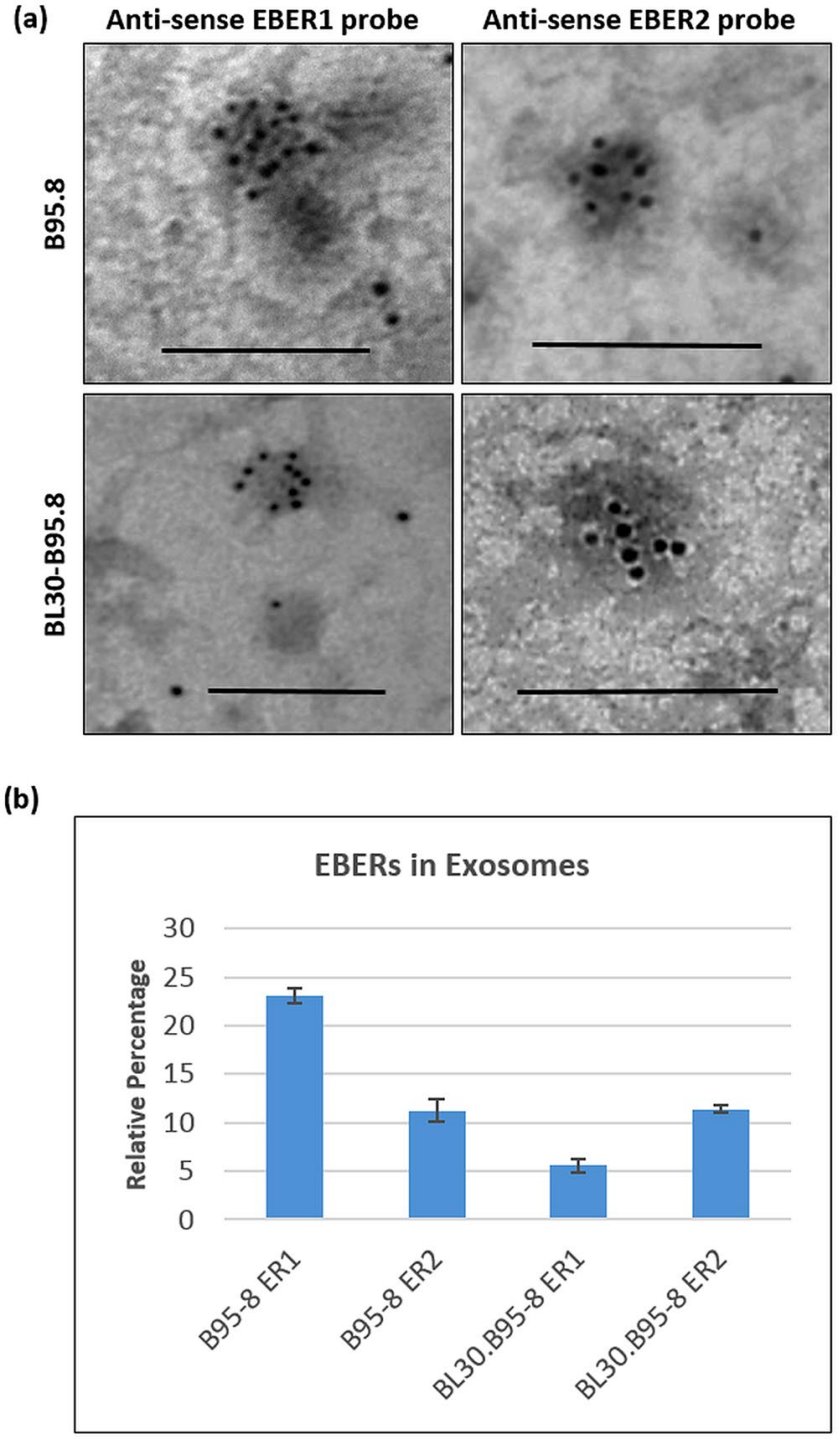
EBER1 and EBER2 are both released in exosomes. (**a**) Electron microscopy *in situ* hybridization for EBER1 and EBER2 revealed that both EBERs were excreted in exosomes of EBV infected cells (B95.8 and BL30-B95.8). (**b**) qRT-PCR was carried out on cDNA generated from 3 µg of the RNA isolated from B95.8 and BL30-B95.8 exosomes. Relative percentage of each EBER (ER1/ER2) was extrapolated from the standard calibration curve established using EBER1 and EBER2 plasmids. The relative percentage of EBER1 present in B95.8 exosomes was found to be twice as much as EBER2. By contrast, in BL30-B95.8 cells, EBER2 appeared to be released in exosomes in higher quantities. Scale bar = 100 nm.

To determine which of the two EBERs are released in exosomes, we performed EM EBER-ISH on exosomes using EBER1 and EBER2 probes separately. The results showed that both EBER1 and EBER2 were released in EBV positive exosomes (Fig. 5a). However, it was challenging to quantitate the relative amounts of each of the two EBERs being excreted. To address this, we performed qRT-PCR on DNase-1 treated RNA extracted from EBV exosomes. Absolute EBER1/2 copy number was determined using a calibration curve generated by performing qRT-PCR on serial dilutions of the EBER1 and 2 plasmids, respectively. Relative percentage of EBER1/2 was estimated by dividing the EBER1/2 copy number in exosomes by the total (cellular plus exosomes) EBER1/2 copy number, respectively. Our estimates indicated that less than 25% of the total EBER1 and EBER2 were released in exosomes. Moreover, it appeared that in B95.8 cells, the amount of EBER1 released in exosomes was more than double compared to EBER2. (Fig. 5b). However, for BL30-B95.8, it appeared that EBER2 rather than EBER1 was released in exosomes at higher amount (Fig. 5b).

### EBER binding protein La is localized in exosomes from EBV infected B-cells

**Figure 6:**
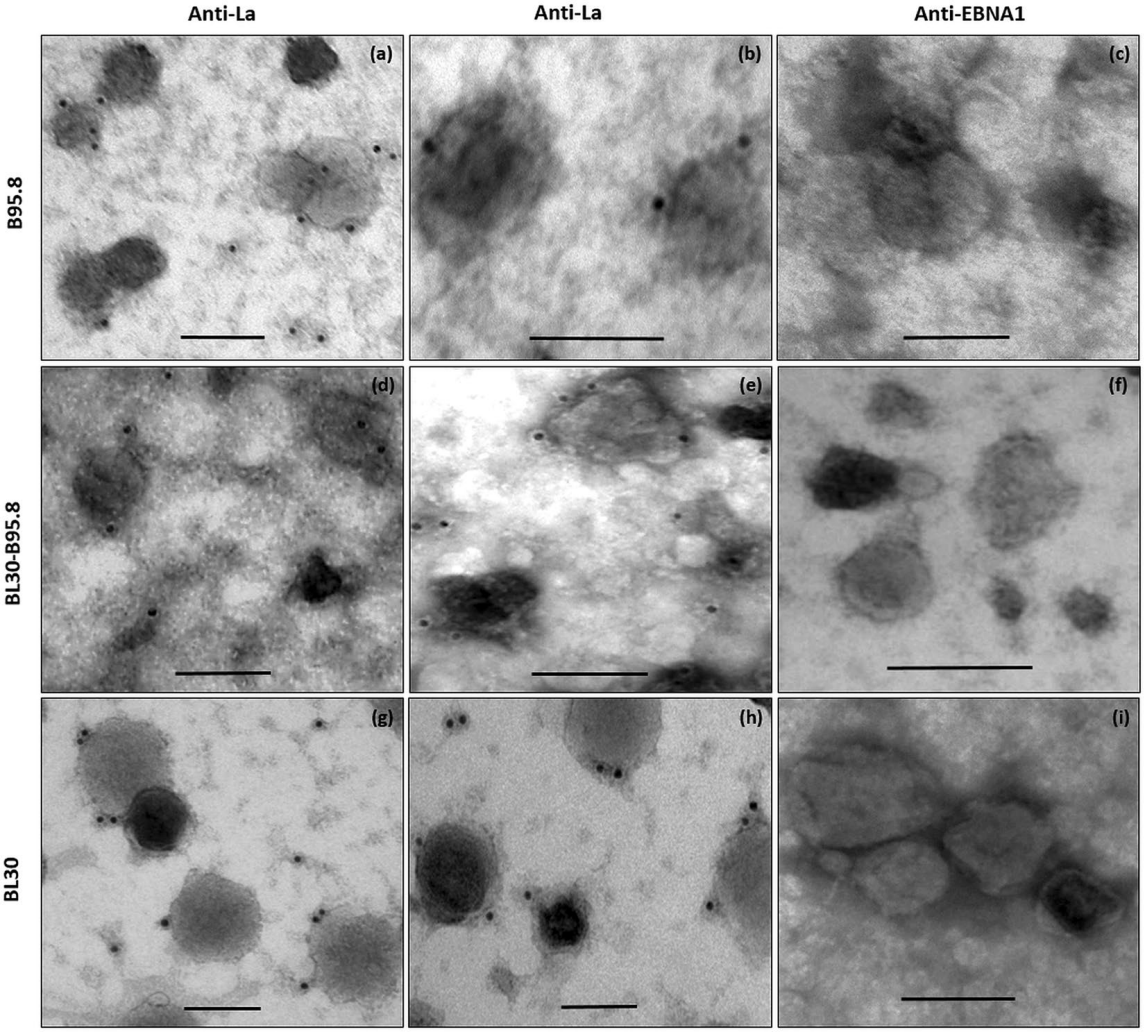
Immuno-electron microcopy staining for La protein in exosomes. Exosomes were fixed overnight in 4% paraformaldehyde and immunostained for La using 10 nm gold labelled secondary antibody. La staining was observed in exosomes isolated from all three cell lines (B95.8, BL30-B95.8, BL30). Immunostaining for EBNA1 was included as a negative control, hence no signals was observed in any of the exosome preparations (**c**,**f** and **e**). Scale bar = 100 nm.

Western blot analysis indicated that La was present in exosomal fractions (Fig. 3b). To determine if La protein was present within exosomes, we performed immuno-EM for La using La specific monoclonal antibodies (Fig. 6). Compared to EBERs, the level of La signal seen in exosomes was relatively low. La is a cellular protein expressed in all types of cells. Thus as expected it was seen in exosomes released from both, EBV infected (B95.8 and BL30-B95.8) and non-infected (BL30) cells (Fig. 6). However, not all exosomes were La positive. Our estimation indicated that less than 40% of exosomes observed under TEM were La positive (Fig. S3). To show that the La staining observed was specific, immuno-EM for EBNA1 was done as a negative control (Fig. 6c). No signals were observed in any of the exosome preparations using anti EBNA1 specific monoclonal antibody.

### EBERs and La are localized together in the same exosomes from EBV infected B-cells

**Figure 7:**
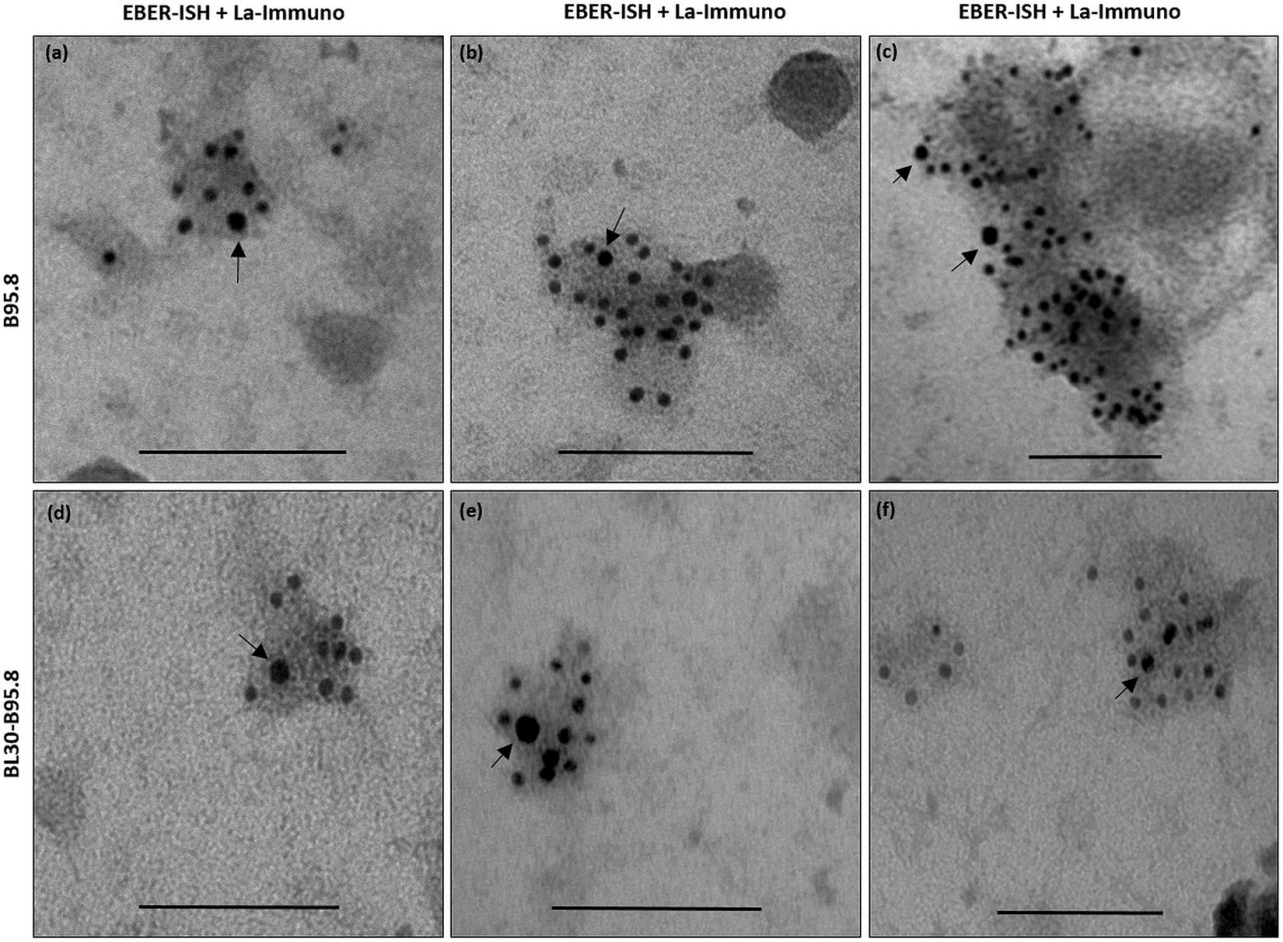
Electron microscopy double staining for EBERs and La in exosomes. Exosomes were isolated from culture supernatant of EBV infected cells and fixed overnight in 4% paraformaldehyde. EM EBER *in situ* hybridization was performed using 5 nm gold labelled secondary antibody and immunostaining for La was performed using 10 nm gold labelled secondary antibody. EBERs and La could be seen co-localized in the same exosomes. Arrows indicate the La staining. Scale bar = 100 nm.

We have previously hypothesized that EBERs are released from EBV infected cells by piggybacking on the La protein^10^. EM EBER-ISH and immuno-EM for La revealed that both EBERs and La are secreted in exosomes from EBV infected cells. However, these data do not show if both, La and EBERs are localized within the same exosomes or are secreted independently. To address this essential question, we performed double staining (EM EBER-ISH and immuno-EM for La) using secondary antibodies conjugated to 5 nm and 10 nm gold particles. Our EM results indicated that both EBERs and La were co-localized in the same exosomes, supporting our hypothesis that EBERs are most probably released out of the exosomes by binding to the La protein (Fig. 7).

## Discussion

EBV can latently persist in memory B cells of the infected host for life. Although in most cases this persistence is harmless to the host, under certain immune perturbations, the virus can play an important role in causing a number of diseases^26,27^. In latently EBV infected cells, the most abundant viral transcripts are the small non coding RNAs (EBERs) and they are present in all forms of viral latency^1^. Why the virus needs to express these non-protein coding small RNAs in such high quantities and what their functions are, remains poorly understood. Previously it was suggested that EBERs do not play any role in the establishment of primary viral infection, or B cell transformation^28^. However, a more recent study reported that EBER deleted virus is significantly less efficient in the transformation of cells compared to the wild type virus^29^. A number of other reports suggest that EBERs contribute to viral oncogenesis by inducing cell proliferation and inhibiting apoptosis^30–32^. Although EBERs are primarily localized in the nucleus^33^, it has been reported that they may also be present in the cytoplasm^12^. This should not come as a surprise because EBERs were first discovered from the sera of SLE patients complexed with the La protein^9^. Furthermore, they were also shown to be present in the cytoplasmic preparations of EBV infected Burkitt’s lymphoma cells^34^. Since EBERs have the ability to bind to a number of cellular proteins that can shuttle back and forth from the nucleus to the cytoplasm, suggests that EBERs could also be present in the cytoplasm, albeit at low levels^35–37^.

It has been suggested that EBERs are released from infected cells via active secretion of La through the exosomal pathway^10^. Moreover, the binding of La to EBERs could also provide stability and protection for EBERs from nuclease degradation. Our previous study revealed that both EBER1 and EBER2 were released from EBV infected cells in exosomal fractions^23^. However, the details of the steps involved in their excretion remained unknown. Furthermore, it was unclear whether EBERs were actually present in exosomes or in extra-exosomal compartments.

In this study we investigated the pathway of EBER release from the nucleus to the exosomes by directly tracking their journey using a novel transmission electron microscopy (TEM) based EBER *in situ* hybridization (EM EBER-ISH). We demonstrate for the first time, the presence of EBERs and La, not only in the nucleus and the cytoplasm of EBV infected B-cell lines, but importantly in the excreted exosomes. It was previously reported that EBERs are mainly confined to the nucleus, whereas the La protein undergoes the nucleocytoplasmic shuttling^11^. Whether EBERs are exported from the nucleus to the cytoplasm by piggybacking on the La protein or by some other mode of excretion remains to be determined. It is also unclear whether EBER excretion in exosomes is dependent on cell type. Our *in vitro* studies indicate that both, EBV infected B-cell lines and EBER-transfected 293-T epithelial cells shed EBER-carrying exosomes^23^. The quantity of EBERs excreted however, could well vary between different cell types, since different cells are known to shed varying degree of exosomes. Moreover, cells at different states could shed different amounts of exosomes, which in turn could also impact the amount of cargo being excreted. Our qRT-PCR analysis indicated that EBER1 was excreted at higher level compared to EBER2, at least in the exosomes isolated from B95.8 cells. The difference could be due to the longer half-life of EBER1 compared to EBER2^38^. Some previous reports have suggested the absence of EBER2 in exosomal fractions^24,25^. This absence could be due to the different methods used for preparation/isolation of exosomes and/or the detection of EBERs. For example, in the current study we faced some challenges in maintaining the overall morphology of the exosomes, primarily due to the harsh conditions of the EM EBER-ISH protocol. Thus, a balance needs to be established between retaining the morphology of the exosomes and detection of EBERs. In addition to EBERs, we were also able to localize the presence of La within exosomes. La is a normal cellular protein expressed in all cell types, independent of EBV infection. We show that at least a fraction of La protein is somehow packaged into MVBs and excreted in exosomes released from EBV infected and non-infected cells. In comparison to EBERs, the level of La signals in exosomes was much lower. Moreover, less than 40% of exosomes appeared to show La signal by TEM. This observation indicates that, unlike CD63, La is not a major constituent of exosomal cargo. Importantly, using double-staining, we were able to demonstrate the presence of both La and EBERs co-localized in the same exosomes, supporting our hypothesis that EBERs are most probably released from infected cells by binding to the La protein. Although, both La and EBERs are present in the same exosomes, definitive proof that La is indeed the main vehicle of transport for EBER excretion is not established from this work. In this regards, our future studies aimed at disrupting the binding of La to EBERs by deleting the La-binding sites in EBERs, could help to resolve this. We are currently exploring this approach, together with siRNA methodology against La to determine if EBER export in exosomes is inhibited in the absence of La. Our observation of lower level of La signals in exosomes compared to EBERs, implies that either multiple copies of EBERs bind to one La molecule or EBERs are excreted in exosomes via alternative routes other than involving La.

Emerging studies in the field of exosomes have suggested that tumor cells release exosomes containing specific cargo that helps in the establishment of pre-metastatic niches^39^. A number of previous studies have indicated that cells infected with EBV can manipulate the local microenvironment by releasing exosomes containing viral and cellular components^18,22^. The contents released in exosomes largely depends on the state of the cell and can thus change over the course of viral infection. How the released EBERs help in EBV pathogenesis needs to be explored. A previous study has shown that EBERs-La complex isolated from the serum of patients with EBV-associated conditions, could induce pro-inflammatory cytokines through TLR3 pathway^24^. More recently, it was shown that the uptake of EBER1 containing exosomes by dendritic cells, results in the induction of IFN-related genes and inflammation mediated tumorigenesis. This effect was largely dependent on the level of EBER1 released in exosomes^40^. Our preliminary data indicates that exosomes released from EBER1 transfected cells induce cell proliferation^19^. It is expected that future studies will address the role and function of EBERs in infected cells and molecular mechanism of their excretion in exosomes.

In conclusion, in this study we have demonstrated using immuno-EM and EM EBER-ISH that EBERs are somehow transported from their primary location in the nucleus to the cytoplasm where they are packaged into MVB. These MVB eventually fuse with the plasma membrane resulting in the release of exosomes containing EBERs. The finding of La protein co-localized with EBERs in the same exosomes, indicates that this cellular protein could be the vehicle of transport for EBERs.

## Materials and Methods

### Cell lines and culture

For the isolation of exosomes, the following EBV cell lines were used: Marmoset EBV-immortalized B-cell line (B95.8)^41^, EBV negative Burkitt’s lymphoma B-cell line (BL30) and its EBV positive counterpart, BL30-B95.8^42^ (kind gift of Professor Martin Rowe, University of Birmingham, UK). All the cell lines were maintained according to the conditions described previously^23^. For exosome isolation, cell lines were grown in 10% exosome depleted FBS which was prepared by ultracentrifugation at 25,000 RPM for 4 hours at 4 °C^43^.

### Isolation of exosomes

Exosomes were isolated from culture supernatants of B95.8, BL30-B95.8 and BL30 cell lines using differential ultracentrifugation^23^. Briefly, supernatant (~80 ml) from each cell line was collected and cellular debris was removed by centrifugation first at 2000 × g for 20 min and then at 10,000 × g for 30 min at 4 °C. Exosomes were pelleted down by ultracentrifugation at 100,000 × g for 70 min followed by a PBS wash. The final exosome pellets were re-suspended in 100 µl of 1X PBS and stored at −80 °C for later analysis.

### Western blotting and RT-PCR

To confirm the presence of exosomes in the harvested pellets, a number of well-known markers for exosomes were examined. The total protein concentration of isolated exosomes was determined using Bradford protein assay (Bio-Rad Laboratories, Hercules, USA). Fifty microgram of exosomal protein was used in each western blot assay. Western blotting using specific monoclonal antibodies for CD63 (clone E-12, Santa Cruz, Cat# sc365604), Flotillin 1 (clone EPR6041, Abcam, Cat# ab133497), CD9 (clone C-4, Santa Cruz, Cat# sc13118) and CD81 (clone B-11, Santa Cruz, Cat# sc166029) was performed under reducing conditions. The presence of EBER binding protein La in exosomal fractions was confirmed using anti La monoclonal antibody (clone B-8, Santa Cruz, Cat# sc166274). Total RNA was isolated from exosomal fractions to investigate the presence of EBER1 and EBER2. RNA was reverse transcribed into cDNA and PCR was performed as described previously^23^.

### Fixation and embedding of cells/exosomes for electron microscopic studies

#### Cells

For the generation of ultra-thin sections for electron microscopy (EM), 5 × 10^6^ cells were fixed with modified Karnovsky’s fixative (4% paraformaldehyde and 0.05% glutaraldehyde, pH7.2) for 4 hours at 4 °C^44^. The cells were then washed with 1X PBS and dehydrated in a series of ascending concentrations of ethanol from 50–100%. Cells were then embedded in LR white resin (London Resin; Agar Scientific, United Kingdom) overnight at 4 °C and then placed in embedding capsules with fresh LR white resin and polymerized by incubating at 50 °C for 2 days. The embedding capsule was removed and the cell blocks were trimmed with razor blades for further processing. Semi-thin (1–2 µm) and ultra-thin (95 nm) sections were cut with Leica Ultracut UC7 ultramicrotome (Leica; Microsystems GmbH, Vienna, Austria) and mounted onto carbon formvar-coated 200 mesh nickel grids (TAAB Laboratories Equipment Ltd, England, UK), ready for immuno-gold staining.

#### Exosomes

Exosomes were fixed by re-suspending the exosome pellet in 100 µl of modified Karnovsky’s fixative for 24 hours at 4 °C. Fifteen microliter of exosome suspension was placed onto carbon formvar-coated 200 mesh nickel grids and incubated at 37 °C for 30 min to allow the membranes to adsorb the exosome suspension.

### Immuno-electron microscopy for CD63 and La on whole mount exosomes

Exosomes were adsorbed on the nickel grids by incubating at 37 °C for 30 min. The grids were washed in 100 µl drop of 1X PBS for 5 min and then transferred into a 100 µl drop of 0.5 M NH_4_CL for 3 min. Blocking was done by placing the grid in 5% BSA for 20 min. Grids were washed and incubated overnight at 4 °C in anti-CD63 monoclonal antibody (ab8219, Abcam, UK) or anti-La monoclonal antibody (sc-166274, Santa Cruz, USA) or anti-EBNA1 monoclonal antibody (MA1-7271, Thermofisher, USA) (negative control). Each antibody was used at 1:25 dilution. Next day, the grids were washed and then incubated in secondary antibody (anti-mouse IgG conjugated to 10-nm gold particles, TAAB, UK) at 1:100 dilution for 2 hours. The grids were washed and fixed in 2.5% aqueous glutaraldehyde for 5 min, then contrasted with 2% uranyl acetate for 5 min and examined using Philips CM10 transmission electron microscope (TEM) (Philips, the Netherland) at 80 K volts.

### Electron microscopy EBER *in situ* hybridization on ultra-thin cell sections and exosomes

#### Preparation of cells for EM

For EM EBER *in situ* hybridization (EM EBER-ISH) on the ultra-thin sections of embedded cells, nickel grids were first jet washed with deionized water and then placed in H_2_O_2_ for 10 min. The grids were washed in deionized water and then incubated in 0.5 M NH_4_Cl in IX PBS for 20 min.

#### Preparation of exosomes for EM

Exosomes were fixed in modified Karnovsky’s fixative and then adsorbed on the nickel grids by incubating at 37 °C for 30 min. The grids were washed in PBS for 5 min and then transferred in a 100 µl drop of 0.5 M NH_4_Cl for 3 min.

#### EM EBER-ISH on cells and exosomes

Digoxigenin (DIG)-labeled EBER probes were diluted in hybridization buffer to a final concentration of 0.2 µg/ml^45^. For the detection of EBER1 or EBER2 individually, DIG-labeled EBER1 and EBER2 probes were used separately. Twenty microliter of the hybridization mix was added on the grids and incubated overnight at 37 °C in a humidified petri dish containing a filter paper soaked in 2X SSC. On the following day, the grids were washed in a 100 µl drop of 0.1X SSC (x6) for 5 min at room temperature and then in a 100 µl drop of 1X PBS (x6) for 5 min. The grids were incubated for 2 hours in anti-DIG antibody (clone D1-22, Sigma, Cat#D8156) at 1:50 dilution. The grids were washed in washing buffer (0.1% BSA in 1X PBS) for 10 min and then incubated with secondary antibody (anti-mouse IgG conjugated to 10-nm gold particles, TAAB, UK) for 1 hour at 1:100 dilution. Grids were washed with 1X PBS and fixed in 2.5% glutaraldehyde for 5 min. The nickel grids were jet washed with deionized water, blot dried, contrasted with 2% uranyl acetate for 5 min and blot dried again before TEM examination.

### Immuno-electron microscopy for La in cells

Immuno-electron microscopy for La (immuno-EM La) was carried out as previously described^46^. Briefly, the grids with cells were jet washed with deionized water and then incubated in aqueous 10% H_2_O_2_ for 10 min, followed by washing in deionized water. Grids were then immersed in 0.5 M NH_4_Cl in 1X PBS for 20 min. Sections were blocked by incubating in 20% normal goat serum for 10 min followed by washing for 5 min in wash buffer (1% bovine serum albumin and 0.1% Tween 20). The nickel grids were incubated for 1 hour with anti-La monoclonal antibody (sc-166274, Santa Cruz, USA) at 1:50 dilution. The grids were washed in wash buffer and incubated in secondary antibody for 1 hour (anti-mouse IgG conjugated to 10-nm gold particles, TAAB, GEM025) at 1:100 dilution. The grids were washed with 1X PBS, fixed in 2.5% aqueous glutaraldehyde for 5 min, and then washed with deionized water and blot dried. The grids were contrasted with 2% uranyl acetate for 5 min and then washed with deionized water. The grids were dried on a filter paper and examined by TEM.

### Double staining for EBERs and La in exosomes

EM EBER-ISH was performed as described above. Following overnight incubation with DIG-labeled EBER probes, the grids were washed and incubated in mouse anti-DIG antibody at 1:50 dilution and rabbit anti-La monoclonal antibody (clone D19B3, Cell Signaling, Cat# 5034S) at 1:25 dilution for 2 hours. The grids were washed and incubated in secondary antibodies; anti-mouse IgG conjugated to 5-nm gold particles and anti-rabbit IgG conjugated to 10-nm gold particles, each at 1:20 dilution for 2 hours. The grids were washed, fixed, contrasted, and subsequently dried on a filter paper and examined by TEM.

### EBER1/2 quantification

RNA was isolated using TRIzol reagent (Invitrogen, USA) from exosomes purified from culture supernatant corresponding to approximately 5 × 10^7^ cells. For quantification, calibration curve was first generated using EBER1 and EBER2 plasmids (pHEBo-E1 and pHEBo-E2) (kind gift of Prof Paul Farrell, Imperial College London, UK). To determine the EBER1/2 copy number, 3 µg of exosomal RNA was transcribed and Syber green qRT-PCR was performed using the Power SYBR^®^ Green PCR Master Mix (Applied biosystems, UK). The details of the primer sequences, concentrations and qPCR conditions used are given in supplementary information (Table S1). Each experiment was repeated twice and all samples were run in triplicates using the Applied Biosystems™ QuantStudio™ 7 Flex Real-Time PCR System.

## Electronic supplementary material

Supplementary information
