# Supplementary material for: Tracking EBV-encoded RNAs (EBERs) from the nucleus to the excreted exosomes of B-lymphocytes
Source: Sci Rep. 2018 Oct 18;8:15438. doi: 10.1038/s41598-018-33758-4 (PMC6193935; doi:10.1038/s41598-018-33758-4)
Supplement: Supplementary file 1 — Supplementary information [file 41598_2018_33758_MOESM1_ESM.pdf]

Supplementary Information

Tracking EBV-encoded RNAs (EBERs) from the nucleus to the excreted exosomes of B-lymphocytes

Waqar Ahmed, Saeed Tariq, Gulfaraz Khan

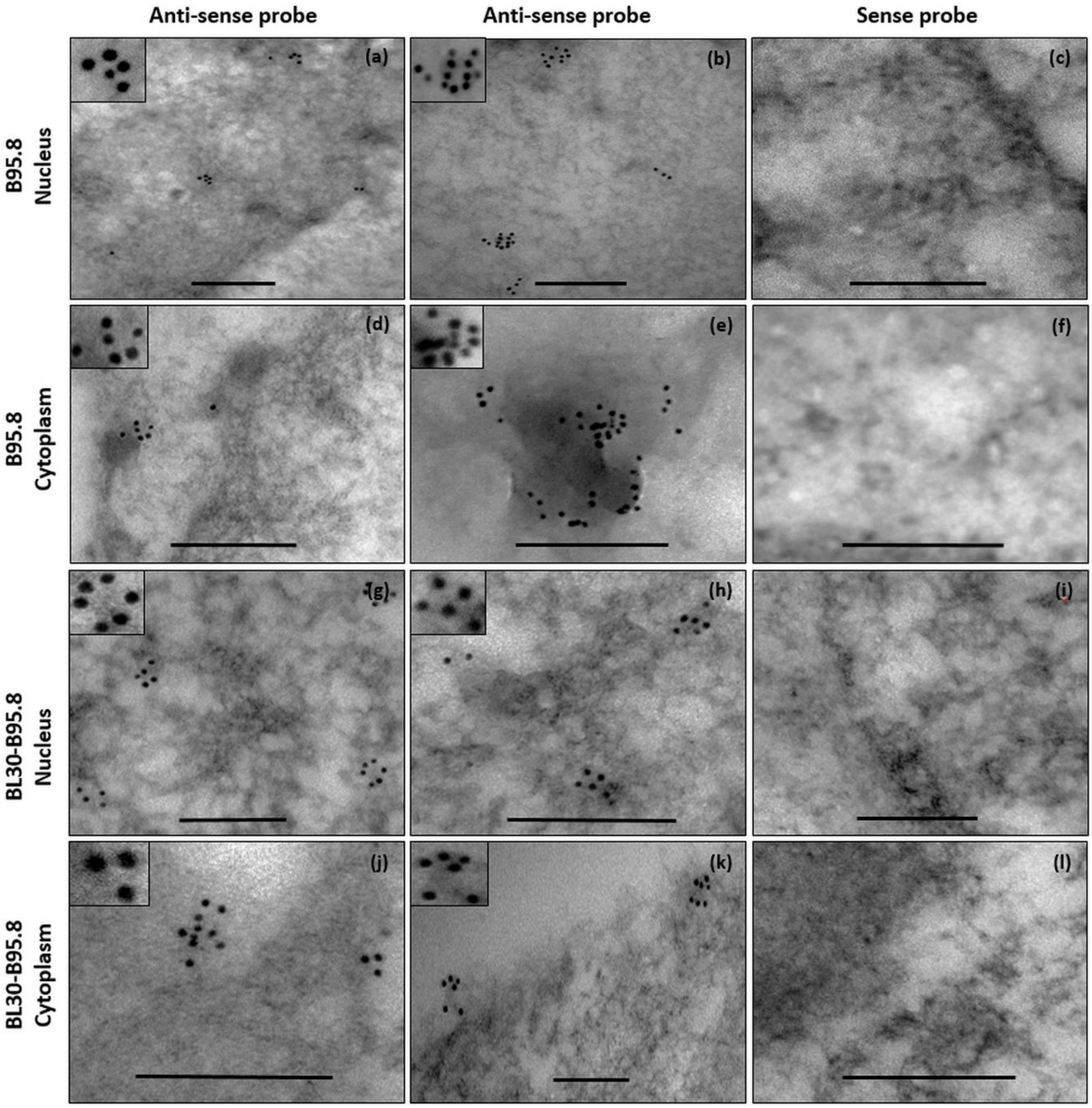

Figure S1: Electron microscopy *in situ* hybridization for EBERs in EBV infected cells. Ultra-thin sections of EBV infected cells were generated and *in situ* hybridization was carried out using antisense and sense EBER probes. . EBER signal was detected using 10nm gold labelled secondary antibody. Specific signals were seen in the nucleus and cytoplasm of B95.8 and BL30-B95.8 cells using antisense probe. No such signal were observed in the nucleus or cytoplasm of the cells using sense probe Scale bar = 200nm

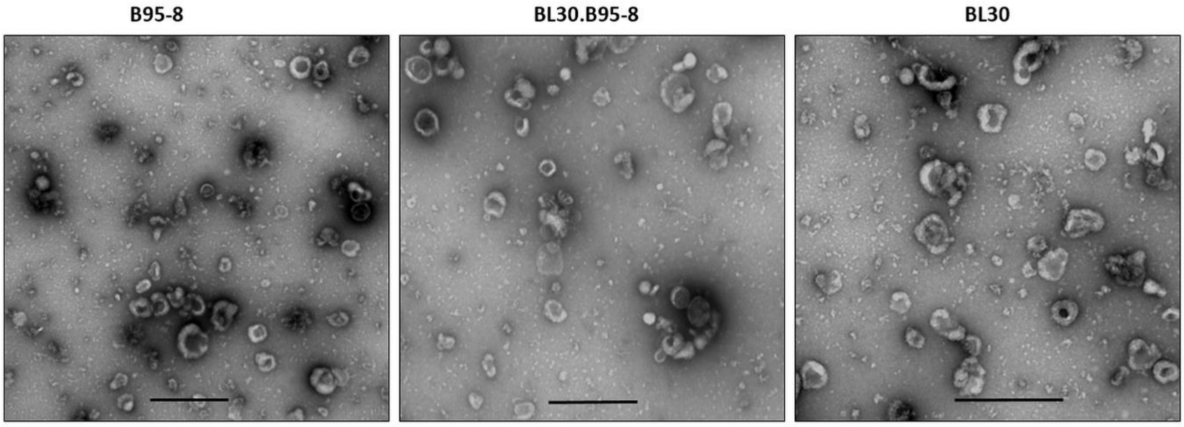

Figure S2: Transmission Electron microscopy on isolated exosomes. Exosomes were isolated using differential ultracentrifugation. Exosomes were fixed on nickel grids by incubating at 37°C for 30 minutes followed by uranyl acetate staining for 5 minutes. Scale bar = 500nm

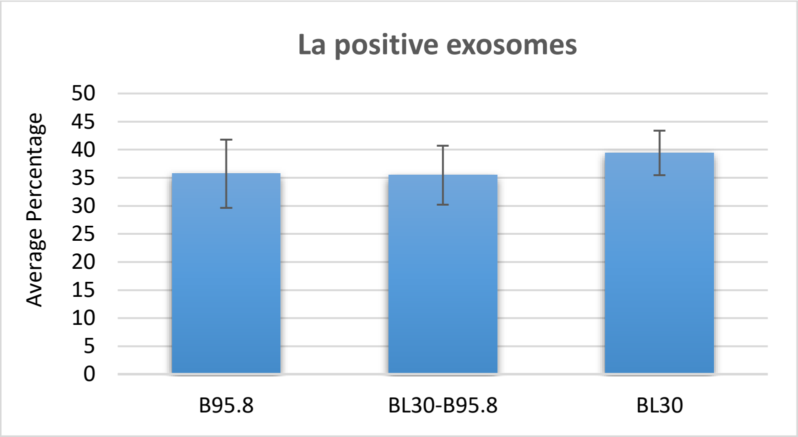

Figure S3: Estimation of La positive exosomes isolated from EBV infected and non-infected cell lines. La immunostaining was performed on fixed exosomes using 10nm gold labelled secondary antibody. The counting of La positive exosomes was performed by choosing 8 different regions on the 200mesh nickel grids at the same magnification (x65,000). The average percentage was calculated by dividing the number of La positive exosomes from the total number of exosomes observed in each field.
